# On Analytical Corrections for Restraints in Absolute Binding Free Energy Calculations

**DOI:** 10.1021/acs.jcim.4c00442

**Published:** 2024-04-19

**Authors:** Stefan Boresch

**Affiliations:** Department of Chemistry, University of Vienna, Währinger Straße 17, A-1090 Vienna, Austria

## Abstract

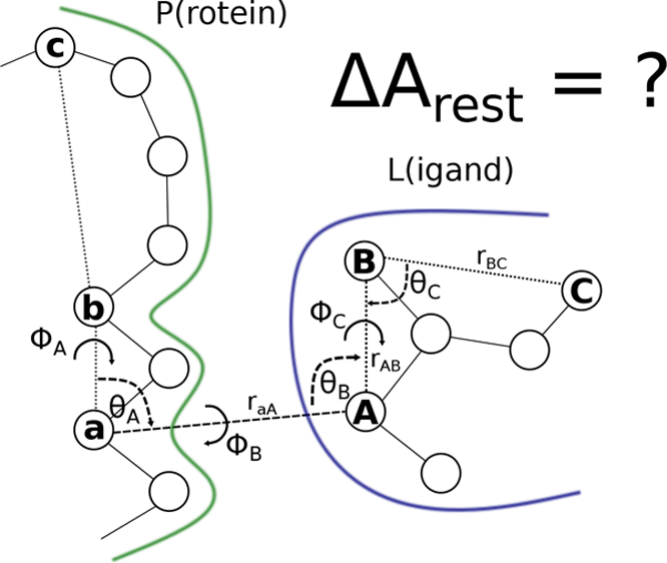

Double decoupling absolute binding free energy simulations require an intermediate state at which the ligand is held solely by restraints in a position and orientation resembling the bound state. One possible choice consists of one distance, two angle, and three dihedral angle restraints. Here, I demonstrate that in practically all cases the analytical correction derived under the rigid rotator harmonic oscillator approximation is sufficient to account for the free energy of the restraints.

## Introduction

The accurate computational prediction of absolute binding free energies (ABFE) would be extremely desirable in both applied and pure medicinal chemistry and biochemistry.^1^ It is not an exaggeration to state that the calculation of ABFEs is considered a “holy grail” of computational chemistry. Already in 1988, Jorgensen and co-workers outlined a thermodynamic cycle which, in principle, made possible ABFE calculations along an alchemical path.^2^ Jorgensen’s approach was used in several studies, e.g., refs (3−5). In 1997, Gilson et al.^6^ showed that to obtain thermodynamically well-defined results, the cycle proposed in ref (2) must be refined, starting with a careful definition of the bound state. As the ligand ceases to interact with the environment (receptor, solvent etc.) its movements relative to the receptor need to remain restricted to regions considered as “bound” in the case of the native complex; see refs (6) and (7) for the details. In practice, the decoupling of protein–ligand interactions has to end with an intermediate state, at which the native interactions between receptor and substrate are replaced by suitable restraints holding the ligand in a position and orientation similar to the native bound state. The chemical potential of the decoupled ligand restrained in such a manner usually does not correspond to the standard state concentration, and this correction has to be accounted for. Similarly, if the rotation of the ligand is restrained, the corresponding free energy cost has to be calculated as well.^6^

Boresch et al. suggested using a set of restraints consisting of one distance, two angle, and three dihedral angle terms, which they referred to as the “virtual bond algorithm” (VBA).^8^ Using suitable approximations, the free energy cost Δ*A*_*rest*_ of removing the VBA restraints can be expressed in closed form (see eq 32 of ref (8) and eq 4 of SI). While several practical issues make the use of VBA restraints challenging in practice,^9^ the need for an intermediate state at which restraints replace the native interactions is undisputed.^7,10^ Recently, Schrödinger extended the capabilities of its FEP+ program to the calculation of ABFEs.^11^ Their approach uses the VBA formalism. However, the free energy cost of removing the restraints is calculated using quite complicated expressions (eqs 4–6 of ref (11)). Other workers using restraints as in or similar to the VBA calculate the free energy correction by numerical quadrature.^12^

Thus, it is of some relevance (i) to describe how one arrives at the much simpler expressions of ref (8) and (ii) to validate under which conditions the resulting approximate expression for Δ*A*_*rest*_ is adequate. The following analysis is not concerned with whether VBA restraints are the best choice in ABFE, but is intended to help workers who use them calculate Δ*A*_*rest*_ as straightforward as possible.

## Theory and Methods

### Background

The classical, configurational partition function of a molecule consisting of *N* atoms is given by
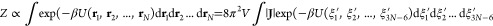
1In eq 1, all prefactors have been omitted, **r**_*i*_ denotes the Cartesian coordinates of the atom *i*, *U* is the system’s potential energy, and β has the usual meaning of 1/*k*_*B*_*T*. The second expression is obtained by separating and integrating over the six external degrees of freedom, corresponding to overall translation and rotation of the system. For a molecule that is free to move through the simulation box, the contribution of the six external degrees of freedom to the partition function *Z* equals 8π^2^*V*, where *V* is the volume of the simulation system. If one operates with standard chemical potentials, as is required for the double-decoupling formalism by Gilson et al.,^6,7^ this system-specific volume *V* has to be replaced by *V*_0_, the volume corresponding to the standard state of choice. E.g., for a standard concentration *c*_0_ = 1 mol/liter, *V*_0_ ≈ 1, 660 Å^3^. The term |**J**| in the second integral in eq 1 is the Jacobian resulting from the coordinate transformation from Cartesian to 3*N* – 6 internal coordinates ξ_*i*_^′^. Since we are not concerned with evaluating the remaining 3*N* – 6-dimensional, configurational integral, the nature of these coordinates and the associated Jacobian are of no import.

**Figure 1:**
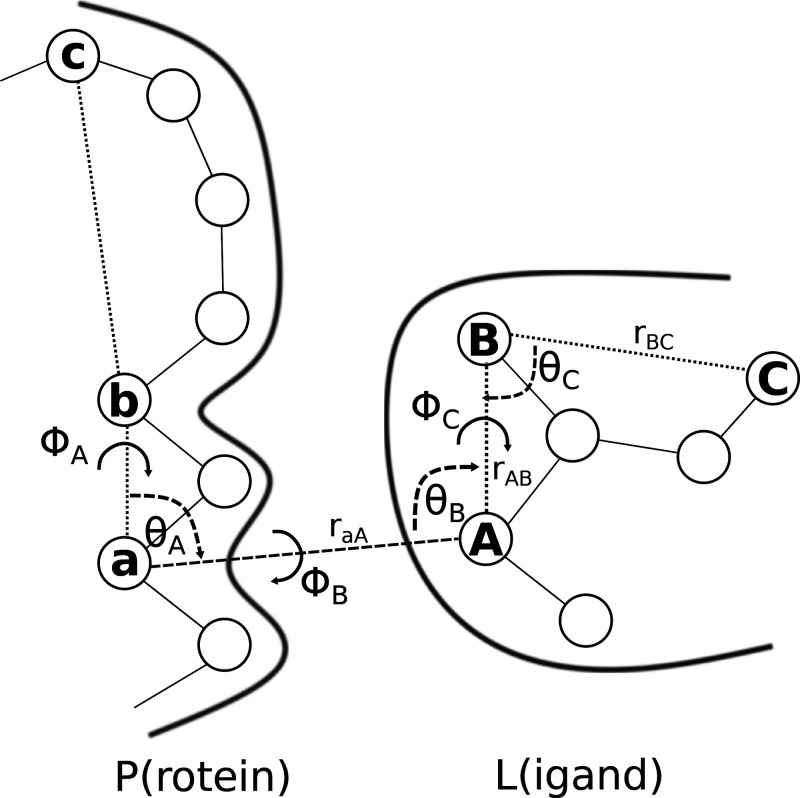
Schematic representation of the six restraints used in the VBA.

In double decoupling ABFE, one needs an intermediate state where the interactions between the protein (P) and the ligand (L) are completely turned off, but restraints are used to maintain L in a position and orientation relative to P that resembles the bound, interacting state. If the restraints are suitably chosen, one can separate a six-dimensional integral *Z*_*rest*_ from the partition function of the ligand, accounting for the contribution of the ligand’s restrained external degrees of freedom. The value of the integral, *Z*_*rest*_, obviously depends on the choice of the restraints. In the VBA algorithm, as well as Schrödinger’s ABFE implementation, the restraints involve three atoms c, b, a in P, and three atoms A, B, C in L. This is shown in Figure 1, which is analogous to Figure 2 of ref (8) and Figure 2 of ref (11). Specifically, there is a distance restraint between a and A (*r*_*aA*_ = *r*), two angle restraints acting on θ_*A*_ and θ_*B*_, respectively, and three dihedral angle restraints acting on the torsions ϕ_*A*_, ϕ_*B*_, and ϕ_*C*_.

### Approximating the Contribution of the VBA Restraints

Let *x*_*i*_ denote the three types of restraints (bond (*r*), angle (θ_*A*_, θ_*B*_) and dihedral angle (ϕ_*A*_, ϕ_*B*_, ϕ_*C*_)), and let each restraint be given by a harmonic potential function, i.e., . The symbol *x*_*i*,0_ denotes the respective target value for the distance, angle, or dihedral angle.

Then the VBA restraint contribution *Z*_*rest*_ to the partition function is given by
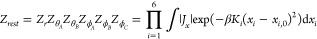
2Here, |*J*_*x*_| is the Jacobian factor for the variable transformation from the Cartesian coordinates to each of the internal coordinates used for the particular restraint. Specifically, for the distance restraint, |*J*_*r*_| = *r*^2^, for the angle restraints, |*J*_θ_| = sin θ, and for the dihedral restraints, |*J*_ϕ_| = 1.^13^

As one sees, eq 2 is the product of six one-dimensional integrals. With computer algebra systems (CAS), analytic expressions can be found easily. This is apparently the route taken by Schrödinger’s team. However, much simpler expressions (as given in ref (8)) can be obtained using two approximations, henceforth referred to as A1 and A2, respectively.

### A1

One assumes that the actual value of *x*_*i*_ will always be close to its equilibrium value *x*_*i*,0_. This is the rigid rotator approximation. Thus, one approximates |*J*_*x*_| ≈ |*J*_*x*_0__|, which makes it possible to take it out of the integral. In other words,
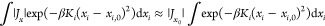
3

### A2

Thus, each of the integrands is a Gauss function. Depending on the type of restraint, there are different limits of integration: 0 to + *∞* for the distance restraint, 0 to π for the angle restraints, and ϕ_0_ ± π for the dihedral angle restraints. If one extends the limits of integration to ± *∞*, then each integral evaluates to
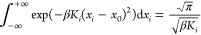
4The configurational integrals approximated by A1 and A2 lead to eq 32 in Boresch et al.^8^ (see also eq 4 in SI).

### Expressions Used by Schrödinger

While Chen et al.^11^ employ the restraints of the VBA and eq 2, they chose, with one exception, not to use the approximations just outlined. This results in the much more complicated expressions for the configurational integrals for the distance, angle, and dihedral angle restraints; see their eqs 4–6 and SI of this work.

The expressions for the distance and the dihedral restraint can be obtained immediately with any modern CAS. We provide example inputs for Mathematica^14^ in SI. Chen et al.’s eq 5, the configurational integral for the angle restraint, contains a ≈ sign, indicating the use of an approximation, the nature of which is not explicitly stated. Their final expression in eq 5 is obtained after applying approximation A2, i.e., extending the limits of integration from (0, π) to ± *∞*, while keeping the Jacobian factor sin θ as part of the integral. Modern CAS can evaluate this improper integral; we provide the Mathematica input in SI.

In fact, the integral with the original limits from 0 to π can be evaluated analytically, either manually or with the help of modern CAS. The resulting expression is somewhat convoluted and contains error functions of complex arguments, but it is not difficult to show that the result is purely real-valued. The necessary derivations are outlined in SI.

### Analyzing the Error of the Approximate Expressions for *Z*_*rest*_

Accurate values for *Z*_*rest*_ are necessary to compute the free energy difference Δ*A*_*rest*_ between the cross-linked protein–ligand “complex”, i.e., the state where native protein–ligand interactions are replaced by the VBA restraints, and the truly noninteracting system, where, in principle, the ligand can move without restrictions. Δ*A*_*rest*_ can, e.g., be written as (cf. the left-hand-side of eq 3 in ref (11)):
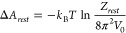
5

Note the use of *V*_0_, i.e., the volume corresponding to the standard state.^6^*Z*_*rest*_ itself is the product of the six configurational integrals, *Z*_*rest*_ = ∏_*i*_^6^*Z*_*i*_ = *Z*_*r*_*Z*_θ_*A*__*Z*_θ_*B*__*Z*_ϕ_*A*__*Z*_ϕ_*B*__*Z*_ϕ_*C*__, cf. eq 2. For each of the *Z*_*i*_, one can utilize either the more rigorous expressions by Chen et al.,^11^ their eqs 4–6 (see also SI), or use the simpler expressions obtained using the two approximations A1 and A2. The latter approach leads to eq 32 of ref (8); see also eqs 4 and 5 in SI. Note that Boresch et al. use Δ*A*_*rest*_ in the direction of *removing* the restraints, whereas Chen et al. consider the *reverse* direction. Of course, one can replace either set of analytical expressions by numerical quadrature of the integrals.^12^

We now want to estimate the error resulting from *Z*_*rest*_^RR^, e.g., the approximate value resulting from the use of approximations A1 and A2, compared to *Z*_*rest*_, an as exact as possible value. From eq 5 it follows that the error resulting from the approximation is given by
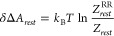
6Thus, a trivial calculation shows that if one requires the value of Δ*A*_*rest*_ to be correct within ±0.1 kcal/mol (*T* = 300 K), then *Z*_*rest*_^RR^ may be too large by 18% or too small by 15.5% relative to the exact (as possible) value of *Z*_*rest*_. Note that a threshold of ±0.1 kcal/mol is almost an order of magnitude smaller than the statistical and systematic errors in ABFE calculations.

Next, let us consider, under what circumstances, approximations A1 and A2 might lead to sizable errors. A1, taking the Jacobian out of the integral, only affects the distance and angle restraints. If the force constants of the restraints are low, then instantaneous values of the distance and/or angle will fluctuate significantly about the target distance/angle. On the one hand, because there are no forces acting on the ligand besides the restraints, it seems a safe approximation that the average distance/angle values will be equal to the target restraint values. On the other hand, Schrödinger’s approach uses a quite low force constant for the distance restraint (1 kcal/(mol Å^2^));^11^ therefore, the use of A1 may be questionable.

The use of A2 changes the limits of integration. For the dihedral restraints, the exact limits of integration run from ϕ_0_ – π to ϕ_0_ + π, i.e., they are symmetrical about the target value of the restraint. Thus, extending them to ± *∞* should not have too large an effect, unless the force constant is extremely weak. The situation is less clear for the distance and angle restraints, where the correct limits of integration are not symmetrical, 0 to *∞* in the distance, and 0 to π in the angle case. Note, however, that for the angles one wants to prevent configurations where the instantaneous angle values are close to 0 or 180°.^9,11^ For this reason, target angles around 90° are preferred, and, second, one needs to set the force constants sufficiently high so that the instantaneous angle values never approach 0 or 180°. These considerations, therefore, suggest that extending the limits of integration in the angle case is acceptable, and this may have been the rationale why Chen et al.^11^ used A2 in this case.

As already mentioned, the Schrödinger approach uses a force constant for the distance restraint of only 1 kcal/(mol Å^2^); i.e., a distance change of 1 Å has an energetic penalty of only 1 kcal/mol. If one ignores the normalizing prefactor, the Boltzmann factor, , can be viewed as a Gaussian probability density function with standard deviation (in Ångstrom) of  at *T* = 300 K. Since in the distance case the upper limit of integration is + *∞*, errors can arise only from the lower limit. The target distance for the restraint is chosen based on the average distance between two (non-hydrogen) atoms in the protein and ligand, respectively. It seems, therefore, unlikely, that the target distance *r*_0_ will be shorter than 3 Å. Assuming an effective σ ≈ 0.55 Å, the difference between the lower limit of integration, *r* = 0, and *r* = *r*_0_ = 3 Å is larger than 5σ ≈ 2.75 Å. In practice, this means that any contribution from extending the lower limit of integration from 0 to – *∞* will be negligible. This is the case even for a low force constant of 1 kcal/(mol Å^2^); obviously, the approximation will become only more accurate if higher force constants are used.

## Results and Discussion

The above considerations suggest that the use of approximations A1 and A2 is unlikely to cause significant errors in most cases. Nevertheless, it is necessary to test this by actual calculations. Three of the restraint sets used as test cases in Table 1, L1A-5, L1A-F, and CL1, were taken from Boresch et al.^8^*Z*_*rest*_ is the restraint contribution to the partition function computed using eqs 4–6 of ref (11); see also eqs 1–3 in SI. Δ*A*_*rest*_ is the corresponding free energy difference when inserting *Z*_*rest*_ in eq 5. *Z*_*rest*_^*RR*^ is the approximate partition function using A1 and A2 (see the expressions for *Z*_*i*_^*RR*^ in eqs 1–3 in SI); the corresponding free energy difference is given by eq 5 in SI. The table also includes values for the restraint free energy using the rigorous expression for the angle configurational integral (eqs 7, 11, and 12 in SI), Δ*A*_*rest*_^*exact*^, as well as using numerical quadrature to evaluate the configurational integrals, Δ*A*_*rest*_^*N*.*Q*.^. The CL1/SCH set has the CL1 target values for the restraints, but the force constants were set to the values suggested by Chen et al.^11^ Finally, set EXTR is intended to test the limits of approximations A1 and A2; all force constants were set to 1 kcal/(mol Å^2^) [1 kcal/(mol rad^2^)]; furthermore, the equilibrium values for the angle restraints were set to 22.5 and 157.5°.

**Table 1:** Restraint Details and Results for Selected Test Cases^a^

System	*K*_*r*_^e^	*r*_0_^f^	*K*_θ_^g^	θ_*A*,0_^h^	θ_*B*,0_^h^	*K*_ϕ_^g^	
L1A-5^b^	5.0	5.10	5.0	67.50	84.50	2.5	
L1A-F^b^	4.0	5.10	8.0	67.50	84.50	5.0	
CL1^c^	20.0	3.48	20.0	89.73	124.14	20.0	
CL1/SCH^d^	1.0	3.48	40.0	89.73	124.14	40.0	
EXTR	1.0	3.00	1.0	22.50	157.50	1.0	

System	*Z*_*rest*_	*Z*_*rest*_^*RR*^	Δ*A*_*rest*_	Δ*A*_*rest*_^*RR*^	|δΔ*A*_*rest*_|	Δ*A*_*rest*_^*exact*^^I^	Δ*A*_*rest*_^*N*.*Q*.^^j^
L1A-5^b^	3.36	3.56	–6.30	–6.27	0.03	–6.30	–6.30
L1A-F^b^	8.49 × 10^–1^	8.78 × 10^–1^	–7.12	–7.10	0.02	–7.12	–7.12
CL1^c^	8.12 × 10^–3^	8.23 × 10^–3^	–9.89	–9.89	0.008	–9.89	–9.89
CL1/SCH^d^	6.62 × 10^–3^	6.51 × 10^–3^	–10.02	–10.03	0.01	–10.02	–10.02
EXTR	8.66	6.64	–5.90	–5.74	0.16	–5.66	–5.66

a Free energies are in kcal/mol. Please see SI for the detailed description of how to obtain the numbers reported below, in particular those already reported by Boresch et al.^8^

b See Tables 2 and 5 of ref (8).

c See Tables 1, 3, and 4 of ref (8).

d As in footnote *c*, but force constants set to the values reported in ref (11).

e Distance restraint force constant in kcal/(mol Å^2^).

f Target value for distance restraint in Å.

g (Dihedral) angle restraint force constant in kcal/(mol rad^2^).

h Target value for angle restraint in degrees.

I Restraint free energy using the exact expression for the configurational integral of the angle restraint (eqs 7, 11, and 12 in SI).

j Restraint free energy obtained using numerical quadrature for all configurational integrals. The NItegrate function of Mathematica was used, but identical results (within machine precision) are found using, e.g., the quad_qag function of Maxima.^15^

As one sees in Table 1, the deviation δΔ*A*_*rest*_ between the rigid rotator harmonic oscillator result Δ*A*_*rest*_^*RR*^ and the more rigorous result Δ*A*_*rest*_ is negligible, i.e., far below 0.1 kcal/mol, for L1A-5, L1A-F, CL1, and CL1/SCH. The CL1/SCH result demonstrates that the use of Δ*A*_*rest*_^*RR*^ (eq 5 in SI) is sufficient even when using a low force constant for the distance restraint. In these four cases, the results Δ*A*_*rest*_ obtained with eq 5 of ref (11) agree within ±0.001 kcal/mol with Δ*A*_*rest*_^*exact*^, using the rigorous expression for the angle configurational integral (eqs 7, 11, and 12 in SI). In the deliberately extreme set EXTR, the error δΔ*A*_*rest*_ increases to 0.16 kcal/mol, which, considering all other sources of errors in ABFE calculations, still seems tolerable. Furthermore, in this case, the Schrödinger correction Δ*A*_*rest*_ also deviates from the rigorous result Δ*A*_*rest*_^*exact*^. Curiously, the exact result is closer to the rigid rotator harmonic oscillator value (−5.74 kcal/mol) than to the value obtained using the expressions by Chen et al. (−5.90 kcal/mol). We repeat that the equilibrium values for the angle restraints of the EXTR set are too close to 0 and 180° and the force constants too weak to guarantee stable simulations;^9,11^ i.e., such restraints should never be chosen in practice. Finally, the restraint correction Δ*A*_*rest*_^*N*.*Q*.^ obtained by numerical quadrature of the configurational integrals agrees perfectly with the exact analytical result, including the EXTR set.

## Summary

The data just presented, together with the theoretical considerations, shows that the rigid rotator harmonic oscillator expression Δ*A*_*rest*_^*RR*^ (eq 32 of ref (8); eq 5 in SI) for the free energy cost of the VBA restraints suffices in the vast majority of cases. It certainly works for the restraint force constants suggested by Chen et al.^11^ The approximation leads to a noticeable error only when choosing force constants and target values for the restraints, which are most likely unsafe in practice (set EXTR in Table 1). In this case, even the more rigorous expressions by Chen et al. are insufficient, and one has to evaluate the configuration integral for the angle restraint without any approximations or by numerical quadrature. The latter provides an accurate alternative to any of the analytical schemes discussed. In particular, the numerical evaluation of the configurational integrals makes it possibly to use other functional forms for the restraints, such as flat-bottom restraints.

## Data Availability

Input for Mathematica/Wolframscript to obtain the results with both approaches as summarized in Table 1 is available at https://github.com/sboresch/abfe_restraints.

## Supporting Information Available

(i) Summarizes the expressions by Chen et al.^11^ and lists the Mathematica commands by which they can be obtained, (ii) provides some hints on how to obtain the numerical results listed in Table 1 and to reproduce the results by Boresch et al.,^8^ and (iii) outlines how to evaluate the configurational integral for the angle restraint exactly (PDF)
