# Supplementary material for: On Analytical Corrections for Restraints in Absolute Binding Free Energy Calculations
Source: J Chem Inf Model. 2024 Apr 19;64(9):3605–9. doi: 10.1021/acs.jcim.4c00442 (PMC11094717; doi:10.1021/acs.jcim.4c00442)
Supplement: Supplementary file 1 — ci4c00442_si_001.pdf [file ci4c00442_si_001.pdf]

Supporting information: On analytical corrections for restraints in absolute binding free energy calculations

Stefan Boresch*

Department of Chemistry, University of Vienna, Währinger Straße 17, A-1090, Vienna, Austria

E-mail: stefan.boresch@univie.ac.at

Phone: +43 (0)1 427752715

Outline how to derive the expressions by Chen et al.[1]

To avoid the reader unnecessary work/derivation, we outline here how Eqs. 4–6 of Ref. 1 can be obtained with a computer algebra system (CAS). The specific commands for Mathematica[2] are provided; see also `https://github.com/sboresch/abfe_restraints`. Furthermore, we give the corresponding expressions using the rigid rotator harmonic oscillator approximation based on the general steps described in the main text. For the nomenclature used, see the main text, in particular Fig. 1. In the Mathematica expressions, a simplified nomenclature is used: $b = \beta = 1/k_B T$; depending on the context, x denotes distance r, angle θ, or dihedral angle ϕ, and x_0 is used for the corresponding equilibrium distance/angle. The force constant is denoted as $k0$ throughout.

Distance restraint:

$$Z_r = \int_0^{\infty} r^2 \exp(-\beta K_r (r - r_0)^2 dr =$$

$$= \frac{1}{4}\left(\frac{\sqrt{\pi}\,(2\beta K_r r_0^2 + 1)\left(\operatorname{erf}\left(r_0\sqrt{\beta K_r}\right) + 1\right)}{(\beta K_r)^{3/2}} + \frac{2r_0 e^{-\beta K_r r_0^2}}{\beta K_r}\right) \quad (1)$$

Eq. 1 (= Eq. 4 of Ref. 1) can be obtained with the single Mathematica command listed as Eq. 1b below. The corresponding rigid rotator harmonic oscillator approximation is

$$Z_r \approx Z_r^{RR} = r_0^2\sqrt{\frac{\pi}{\beta K_r}} \quad (1a)$$

```
Integrate[x^2 Exp[-b k0 (x - x0)^2], {x, 0, +Infinity},
        Assumptions -> b > 0 && k0 > 0 && x0 > 0]                (1b)
```

Angle restraint: Here, Chen et al. also use an approximation,[1] i.e., changing the limits of integration from $(0, \pi)$ to $\pm\infty$.

$$Z_\theta = \int_0^{\pi} \sin\theta \exp(-\beta K_\theta (\theta - \theta_0)^2 d\theta \approx \int_{-\infty}^{+\infty} \sin\theta \exp(-\beta K_\theta (\theta - \theta_0)^2 d\theta = \quad (2)$$

$$\frac{\sqrt{\pi}\exp\left(-\frac{1}{4\beta K_\theta}\right)\sin(\theta_0)}{\sqrt{\beta K_\theta}}$$

$$Z_\theta \approx Z_\theta^{RR} = \sin\theta_0\sqrt{\frac{\pi}{\beta K_\theta}} \quad (2a)$$

The following Mathematica command leads immediately to the result given in Eq. 2. A full derivation is given in the Section *Exact solution for the angle configurational integral* below.

```
Integrate[Sin[x] Exp[-b k0 (x - x0)^2], {x, -Infinity, +Infinity},
        Assumptions -> b > 0 && k0 > 0 && x0 > 0]                (2a)
```

Dihedral angle restraint Here, since there is no Jacobian factor, the only approximation needed to obtain Z_ϕ^{RR} is extending the limits of integration to $\pm\infty$.

$$Z_\phi = \int_{\phi_0-\pi}^{\phi_0+\pi} \exp(-\beta K_\phi(\phi-\phi_0)^2 d\phi = \frac{\sqrt{\pi}\,\mathrm{erf}\left(\pi\sqrt{\beta K_\phi}\right)}{\sqrt{\beta K_\phi}} \approx \sqrt{\frac{\pi}{\beta K_\phi}} = Z_\phi^{RR} \tag{3}$$

For completeness, the Mathematica expression to obtain the exact expression for Z_ϕ:

```
Integrate[Exp[-b k0 (x - x0)^2], {x, phi-Pi, phi+Pi},
    Assumptions -> b > 0 && k0 > 0 && x0 > 0]                (3a)
```

Hints on obtaining the numerical results

The numerical examples given in the main text are based on some results/tests provided in Ref. 3. When operating with the expressions provided by Boresch et al. (specifically, their Eq. 32), there are some pitfalls to keep in mind. Therefore, we provide a detailed description of how to reproduce the numbers reported in Table 1 of the main text, as well as the values reported in Tables 3–5 of Ref. 3. Example input for Mathematica can be found at `https://github.com/sboresch/abfe_restraints`.

(1) We reproduce here Eq. 32 of Ref. 3 with minor adjustments of the nomenclature

$$\Delta A^{RR}_{rest,BOR2003} = -k_B T \ln\left[\frac{8\pi^2 V_0}{r^2_{a,A,0}\sin\theta_{A,0}\sin\theta_{B,0}}\frac{(K'_r K'_{\theta_A} K'_{\theta_B} K'_{\phi_A} K'_{\phi_B} K'_{\phi_C})^{1/2}}{(2\pi k_B T)^3}\right] \tag{4}$$

Eq. 4 was derived using $U(x) = \mathbf{K'/2}(x-x_0)^2$ as the expression for the harmonic restraint potential function. If one does not include the factor 1/2, i.e., $U(x) = K(x-x_0)^2$, this leads to a slightly modified expression for the restraint contribution

$$\Delta A^{RR}_{rest} = -k_B T \ln\left[\frac{8\pi^2 V_0}{r^2_{a,A,0}\sin\theta_{A,0}\sin\theta_{B,0}}\frac{(K_r K_{\theta_A} K_{\theta_B} K_{\phi_A} K_{\phi_B} K_{\phi_C})^{1/2}}{(\pi k_B T)^3}\right] \tag{5}$$

Eq. 5 is more in line with the internal workings of CHARMM,[4] which uses $U(x) = K(x-x_0)^2$

for harmonic potentials, i.e., the factor 1/2 is absent. The force constants reported in Ref. 3 are the values for the CHARMM internal functional form *without* the factor 1/2. Therefore, upon inserting numbers in Eq. 4 (Eq. 32 of Ref. 3), one has to double each force constant since $K' = 2K$. For example, when using Eq. 4, one has to employ force constants of 40 kcal/(mol Å^2) [40 kcal/(mol rad^2)], i.e., 2×20 kcal/(mol Å^2) [2×20 kcal/(mol rad^2)], to reproduce the CL1 results in Tables 3 and 4 of Ref. 3 and in Table 1 of the main text. By contrast, when using the RR approximations given in Eqs. 1–3 above, which lead to Eq. 5, one can use the force constants of 20 kcal/(mol Å^2) [20 kcal/(mol rad^2)] "as is" to obtain the CL1 result.

(2) The CL1–CL3 examples of Ref. 3 strictly used harmonic restraints, so depending on the working equation employed, force constants may need to be multiplied by two as just described. However, in the second set of results reported by Boresch et al. (tyrosine-tyrosyl-tRNA-synthetase model calculations, see Tables 2 and 5 of Ref. 3), the three dihedral angle degrees of freedom were restrained with CHARMM's dihedral restraint facility. The functional form used was $U(\phi) = k'_\phi(1 - \cos(\phi - \phi_0))$. To use the harmonic oscillator approximation, the first order Taylor approximation was used, i.e.,

$$U(\phi) = k'_\phi(1 - \cos(\phi - \phi_0)) \approx k'_\phi/2(\phi - \phi_0)^2 = k_\phi(\phi - \phi_0)^2$$

with $k_\phi = k'_\phi/2$. Note that because of the Taylor formula, the factor 1/2 appears in the harmonic approximation for the dihedral restraint. In these cases, the situation is reversed. Using Eq. 4, the multiplication by 2 must not be applied to the dihedral force constants. Consider, e.g., restraint L1A-F, Table 5 of Ref. 3, for which we also report results in Table 1 of the main text. Here, different force constants for the individual terms were used; that is, the distance restraint had a force constant of 4 kcal/(mol Å^2), the two angle restraints had a force constant of 8 kcal/(mol rad^2), and the three dihedral angle restraints had a force constant of 10 kcal/(mol rad^2). When calculating ΔA^{RR}_{rest} using Eq. 4 (Eq. 32 of Ref

3), the distance and angle force constants need to be doubled, whereas the dihedral force constants can be used as is. By contrast, when utilizing the expressions derived here (i.e., Eq. 5 above), the force constants for the distance and angle restraints can be used as is, but one has to scale the dihedral force constants by 1/2 ($k'_\phi = 10$ kcal/(mol rad^2) corresponds to $k_\phi = k'_\phi/2 = 5$ kcal/(mol rad^2)).

Exact solution for the angle configurational integral Z_θ

For completeness, we derive here the exact expression for the configurational integral of the angle restraint. Modern CAS will provide closed solutions for both the antiderivative, and for the full integral from 0 to π, but the resulting expressions are difficult to understand. Furthermore, the result contains error functions of complex arguments, suggesting that the configurational integral might be complex. Breaking the problem into a series of simpler integrals and exploiting properties of the error function, one not only obtains a more readable expression but can show that the result is purely real.

We start with the substitution $t = \theta - \theta_0$, i.e.,

$$Z_\theta = \int_0^\pi \sin(\theta)\exp(-\beta K_\theta(\theta-\theta_0)^2 d\theta = \int_{-\theta_0}^{\pi-\theta_0} \sin(t+\theta_0)\exp(-\beta K_\theta t^2)dt \tag{6}$$

and now focus on the antiderivative

$$I = \int \sin(t+\theta_0)\exp(-\beta K_\theta t^2)dt = \cos\theta_0 \underbrace{\int \sin t \exp(-\beta K_\theta t^2)dt}_{I1} + \sin\theta_0 \underbrace{\int \cos t \exp(-\beta K_\theta t^2)dt}_{I2} \tag{7}$$

Using a CAS, e.g., Mathematica[2] with the rule-based integration system[5] loaded,[1] one finds

[1]Mathematica has no difficulties evaluating the integrals, but provides the answer in a form which is more difficult to proceed.

for the two integrals on the right

$$\text{I1} = i\frac{\sqrt{\pi}}{4\sqrt{\beta K_\theta}}\exp(-1/(4\beta K_\theta))\left[\text{erf}\left(\sqrt{\beta K_\theta}t + \frac{i}{2\sqrt{\beta K_\theta}}\right) - \text{erf}\left(\sqrt{\beta K_\theta}t - \frac{i}{2\sqrt{\beta K_\theta}}\right)\right] \quad (8)$$

$$\text{I2} = \frac{\sqrt{\pi}}{4\sqrt{\beta K_\theta}}\exp(-1/(4\beta K_\theta))\left[\text{erf}\left(\sqrt{\beta K_\theta}t + \frac{i}{2\sqrt{\beta K_\theta}}\right) + \text{erf}\left(\sqrt{\beta K_\theta}t - \frac{i}{2\sqrt{\beta K_\theta}}\right)\right] \quad (9)$$

Note that the arguments of the error functions are the complex conjugate to each other. One basic property of the complex error function (see 7.1.10 of Ref. 6), is the identity

$$\text{erf}(\bar{z}) = \overline{\text{erf}(z)} \quad (10)$$

where the bar denotes the complex conjugate. Because of this, the difference and sum of the two error functions in the expressions for I1 and I2 reduce to $2i \times \text{Im}\left[\text{erf}\left(\sqrt{\beta K_\theta}t + \frac{i}{2\sqrt{\beta K_\theta}}\right)\right]$ and $2 \times \text{Re}\left[\text{erf}\left(\sqrt{\beta K_\theta}t + \frac{i}{2\sqrt{\beta K_\theta}}\right)\right]$, respectively, where Re() and Im() denote the real and imaginary part of a complex number. Inserting this in Eqs. 8 and 9 leads to the simpler expressions

$$\text{I1} = -\frac{\sqrt{\pi}}{2\sqrt{\beta K_\theta}}\exp(-1/(4\beta K_\theta))\ \text{Im}\left[\text{erf}\left(\sqrt{\beta K_\theta}t + \frac{i}{2\sqrt{\beta K_\theta}}\right)\right] \quad (11)$$

$$\text{I2} = \frac{\sqrt{\pi}}{2\sqrt{\beta K_\theta}}\exp(-1/(4\beta K_\theta))\ \text{Re}\left[\text{erf}\left(\sqrt{\beta K_\theta}t + \frac{i}{2\sqrt{\beta K_\theta}}\right)\right] \quad (12)$$

which in turn can be used to express the full antiderivative I (Eq. 7).

To evaluate the definite integral Z_θ one still has to calculate the error function for a complex argument, but the above derivation shows that the final result is real, i.e., has no imaginary component. Furthermore,

$$\lim_{a\to\pm\infty} \text{erf}(a + i\,b) = \pm 1 \quad (13)$$

so if the limits of integration are extended from 0 to π to $\pm\infty$, the imaginary part of the error function vanishes and the real part gives +1 for $+\infty$, -1 for $-\infty$. Hence, we obtain immediately

$$Z_\theta \approx \int_{-\infty}^{+\infty} \sin\theta \exp(-\beta K_\theta(\theta-\theta_0))^2 d\theta$$
$$= \Big[\cos\theta_0 \text{ I1} + \sin\theta_0 \text{ I2}\Big]_{\infty}^{+\infty} = \sin\theta_0 \frac{\sqrt{\pi}}{\sqrt{\beta K_\theta}} \exp(-1/(4\beta K_\theta)) \quad (14)$$

which is Eq. 5 of Ref. 1. This approximation can be derived in various ways more easily; any modern CAS gives the result directly when using $\pm\infty$ as limits of integration. Nevertheless, it is a consistency check that the result can also be obtained in a straightforward manner from the exact antiderivative.

Two additional observations follow from Eq. 7 in combination with Eqs. 11 and 12. First, if the equilibrium angle θ_0 is near 90° ($\pi/2$), then $\cos\theta_0$ is close to 0 and most of the contributions to Z_θ will come from the $\sin\theta_0 \times$ I2 term in Eq. 7. Second, compared to the exact expression Eq. 6, both the RR approximation and Schrödinger's approximation (cf. Eq. 2) are incorrect for $\theta_0 = 0$ and $\theta_0 = \pi$. Both approximations give zero in these cases, whereas the exact expression leads to a non-zero value. In practice, however, target angle values near 0 or π need to be avoided anyway to avoid singularities in the forces,[1,7] so in practice this limitation of both approximate expressions can be safely ignored.

References

(1) Chen, W.; Cui, D.; Jerome, S. V.; Michino, M.; Lenselink, E. B.; Huggins, D. J.; Beautrait, A.; Vendome, J.; Abel, R.; Friesner, R. A.; Wang, L. Enhancing Hit Discovery in Virtual Screening through Absolute Protein–Ligand Binding Free-Energy Calculations. *Journal of Chemical Information and Modeling* **2023**, *63*, 3171–3185.

(2) Wolfram Research, Inc., Mathematica, Version 13.3, `https://www.wolfram.com/`

`mathematica`, Champaign, IL, 2023.

(3) Boresch, S.; Tettinger, F.; Leitgeb, M.; Karplus, M. Absolute Binding Free Energies: A Quantitative Approach for Their Calculation. *The Journal of Physical Chemistry B* **2003**, *107*, 9535–9551.

(4) Brooks, B. R. et al. CHARMM: The biomolecular simulation program. *J. Comput. Chem.* **2009**, *30*, 1545–1614.

(5) Rich, A.; Scheibe, P.; Abbasi, N. Rule-based integration: An extensive system of symbolic integration rules. *Journal of Open Source Software 3*, 1073.

(6) Abramowitz, M.; Stegun, I. A. *Handbook of Mathematical Functions with Formulas, Graphs, and Mathematical Tables*, ninth dover printing, tenth gpo printing ed.; Dover: New York, 1964.

(7) Clark, F.; Robb, G.; Cole, D. J.; Michel, J. Comparison of Receptor–Ligand Restraint Schemes for Alchemical Absolute Binding Free Energy Calculations. *Journal of Chemical Theory and Computation* **2023**, *19*, 3686–3704.
